# Garlic Extract Participates in the Proliferation and Apoptosis of Nonsmall Cell Lung Cancer Cells Via Endoplasmic Reticulum Stress Pathway

**DOI:** 10.1155/2023/4025734

**Published:** 2023-02-06

**Authors:** Deguang Pan, Mingjun Zheng, Jin Liu, Ze Sun, Xiulian Shi

**Affiliations:** ^1^Department of Cardiothoracic Surgery, Chun'an County People's Hospital, Hangzhou, Zhejiang, China; ^2^Department of Orthopedics, Chun'an County People's Hospital, Hangzhou, Zhejiang, China; ^3^Department of Emergency, Chun'an County People's Hospital, Hangzhou, Zhejiang, China; ^4^Department of Medical Education, Chun'an County People's Hospital, Hangzhou, Zhejiang, China

## Abstract

**Objective:**

To investigate the effect of garlic extract (GE) on the proliferation and apoptosis of cell lines A549 and H1299 in lung cancer (LC).

**Methods:**

A549 and H1299 cells with well-developed logarithmic growth were added with GE at a concentration of 0 *μ*g/ml, 25 *μ*g/ml, 50 *μ*g/M, 75 *μ*g/ml, and 100 *μ*g/ml, respectively. The inhibition of A549 cell proliferation was detected using CCK-8 after cultured for 24 h, 48 h, and 72 h. The apoptosis of A549 cells was analyzed via flow cytometry (FCM) after 24 h of cultivation. In vitro migration of A549 and H1299 cells was determined by cell wound scratch assay after 0 h and 24 h culture. The caspase-3 and caspase-9 protein expression levels in A549 and H1299 cells were evaluated through western blot after 24 h of cultivation.

**Results:**

Colony formation and EdU assays revealed that Z-ajoene could inhibit cell viability and cell proliferation in NSCLC cells. After 24 h culture, there was no significant difference in the proliferation rate of A549 and H1299 cells with different GE concentrations (*P* > 0.05). A remarkable proliferation rate difference emerged between A549 and H1299 cells with different GE concentrations after 48 and 72 hours of cultivation. The proliferation rate of A549 and H1299 cells in the experiment group was significantly lower than that in the control group. With an elevated level of GE concentration, the proliferation rate of A549 and H1299 cells decreased (*P* < 0.05) while the apoptotic rate increased continuously.

**Conclusion:**

GE could exert toxic effects on A549 and H1299 cells, inhibit cell proliferation, promote apoptosis, and attenuate cell migration. Meanwhile, it might induce apoptosis of A549 and H1299 cells through the caspase signal pathway, which is positively correlated with the mass action concentration and is expected to be a new drug for LC treatment.

## 1. Introduction

Lung cancer remains the most common malignancy and the leading cause of cancer-related death worldwide over the past decades [1], and nonsmall cell lung cancer (NSCLC) accounts for over 80% of all lung cancer diagnoses [2, 3]. Despite the advances in management and diagnostics for NSCLC, the overall survival (OS) of NSCLC patients has not been significantly improved, with the 5-year OS rate of less than 20% [4–6]. Drug resistances, undesirable side effects of chemotherapy, and high metastatic rates are significant obstacles to successful treatment of NSCLC [7] as well as insufficient efforts in disease prevention and early diagnosis and integrated therapies for early remission, though enormous, which does not support a reduced survival rate [8]. It is still imperative to develop more effective drugs and more sensitive diagnostics for timely prevention and intervention to improve NSCLC survival.

ER is the main site for protein synthesis and folding in cells, and is responsible for other basic cellular activities, including intracellular protein maturation and translocation [9]. ER stress (ERS) is initiated when ER homeostasis disturbance results in unfolded/misfolded protein accumulation in the ER lumen. In this process, ERS triggers a series of accommodative mechanisms, known as the unfolded protein response (UPR), including attenuation of translation, elevated expressions of ER chaperones and associated proteins, and degradation of unfolded/misfolded proteins by a quality-control system [10]. However, once the restoration of ER function fails, apoptotic signalings will be induced by ERS [11, 12].

**Figure 1:**
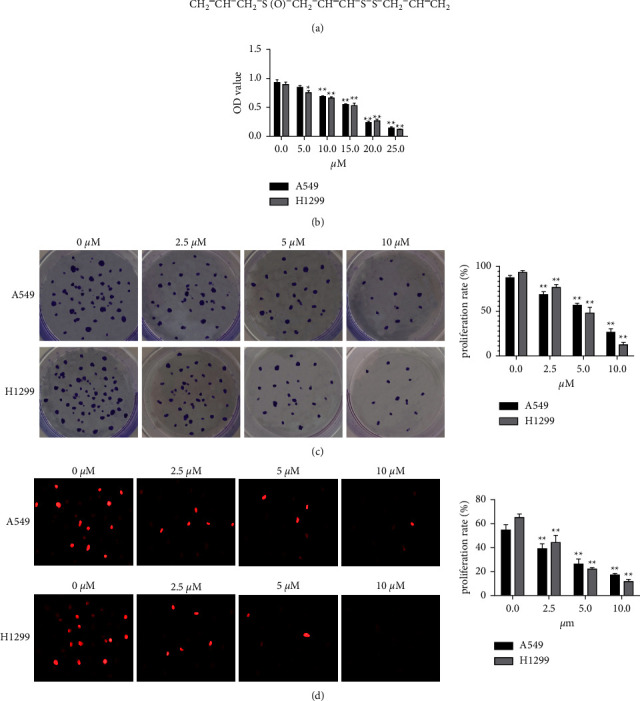
Z-Ajoene inhibits cell viability and cell proliferation in NSCLC cells. (a) Z-ajoene in garlic extracts. (b) A549 and H1299 cells were treated with Z-ajoene for 72 h and subjected to the CCK-8 assay. (c and d) Colony formation and EdU assays revealed suppressed cell proliferation in A549 and H1299 cells.

Garlic (*Allium sativum* L., *Amaryllidaceae*) is one of the oldest known spices and a widely used flavoring agent that has been well utilized to treat numerous ailments over thousands of years and is also considered one of the most powerful chemopreventive and anticancer foods (20). Potential therapeutic properties, such as antithrombotic [13], lipid-lowering [14], anti-oxidative [15], and anti-hypercholesterolemia activities [16], have been studied. In the present study, we reported the effects of Z-ajoene (Z-4, 5, 9-trithiadodeca- 1, 6, 11-triene 9-oxide) (Figure 1(a)) found in crushed garlic on NSCLC cell behaviors and ERS. The anti-inflammatory [17], anti-oxidant, [18], and antifatty liver activities of ajoene, a mixture of E− and Z-isomers, have been previously reported [19–21].

## 2. Materials and Methods

### 2.1. Ajoene Extract and Z-Ajoene from Garlic

Garlic (2 kg) was bought in the Korean retail market, divided into three parts, put into three different plastic buckets, and soaked in different concentrations of alcohol solution to extract the corresponding ingredients. Next, after incubating at room temperature for 1 hour, it was extracted with ethyl acetate (at 60°C for 8 hours). The ethyl acetate extract was evaporated in vacuo to make ajoene extract for *in vitro* experiments. The observed content of Z-ajoene determined by high-performance liquid chromatography (HPLC) was 11.1% (w/w), which was subsequently isolated and purified by repeated column chromatography. As described previously, z-ajoene extract with a purity greater than 98% was obtained [22].

### 2.2. Cell Culture, Treatment, and Transfection

Human NSCLC A549 and H1299 cell lines were obtained from the American Type Culture Collection (ATCC, Manassas, VA, USA). The cells were cultured in RPMI-1640 medium supplemented with 10% FBS in a humidified environment after resuscitation, and the medium was replaced with a fresh medium every other day. The cells were divided into five groups: control, Z-ajoene, Z-ajoene + DLG1-specific shRNA (sh-DLG1), Z-ajoene + YAP-specific shRNA (sh-YAP), and Z-ajoene + sh-DLG1 + sh-YAP. Those in the four treatment groups were incubated with Z-ajoene for 72 h at 37°C. Three treatment groups were transfected with sh-DLG1 and/or sh-YAP using Lipofectamine 3000 reagent (Life Technologies, Carlsbad, CA, USA) to knock down DLG1 and/or YAP. The cells transfected with sh-RNA-NC were used as negative controls (GenePharma, Guangzhou, China). The target sequences for sh-DLG1 and sh-YAP are 5′-AGC GAU GGU CCA UTC UUG CAA-3′ and 5′-GGG UCT UGC AUT UGC ACU ATT-3′, respectively.

### 2.3. Cell Counting Kit-8 Assay

Cell viability of each group was detected using the CCK-8 assay (Beyotime, Beijing, China). The cells at an appropriate concentration were inoculated in 96-well plates and treated accordingly. CCK-8 solution was added to each well, followed by two h of incubation in the dark. The optical density was measured at 450 nm.

### 2.4. Colony Formation Assay

Cells (1,000 cells/well) were seeded in a 6-well plate and incubated for two weeks. Clones were fixed with methanol, stained with 0.1% crystal violet for four h at room temperature, and counted under a light microscope (Olympus, Tokyo, Japan).

### 2.5. 5-Ethynyl-2′-Deoxyuridine (EdU) Assay

Cell proliferation was measured using an EdU assay. A total of 2 × 10^5^ cells were transferred into 24-well plates and allowed to adhere overnight. After transfection, the cells were incubated with 100 *μ*L EdU for two h, fixed with 4% paraformaldehyde for 30 min, and stained using a Cell-LightTM EdU Apollo®488 In Vitro Imaging kit (Guangzhou RiboBio Co., Ltd.) according to the manufacturer's instructions.

### 2.6. Cell Apoptosis Assay

Flow cytometry was conducted to detect cell apoptosis. The cells (1 × 10^6^ cells/well) were seeded in 6-well plates. They were harvested after 24 h of treatment and stained with an annexin-V fluorescein isothiocyanate (FITC) and propidium iodide (PI) apoptosis detection kit (Invitrogen, USA) to detect cell apoptosis using a FACScalibur flow cytometer (BD Biosciences, CA, USA).

### 2.7. Quantitative Real-Time PCR

Total RNA from cell samples was extracted using the TRIpure isolation reagent (Invitrogen, USA), and reverse transcription was performed using a PrimeScript RT kit (TaKaRa, Japan). After the master mix was prepared, DLG1 and YAP mRNA expression levels were detected using a real-time PCR assay with SYBR green, normalized to internal control ACTB.

### 2.8. Western Blot Analysis

Total proteins in tissues or cells were cleaved with RIPA lysate buffer (Beyotime Inc., China), including benzosulfonyl fluoride (Beyotime Inc., China) and protease inhibitor cocktail (Beyotime Inc., China). SDS-PAGE was conducted to isolate 20 mg of protein and transfer it to the PVDF membrane (Millipore Inc., USA, No. sc-27655). The membrane was incubated overnight with the primary antibody monoclonal Bax antibody (1 : 1000, cat. no. YZ-29450 N), monoclonal cleaved caspase three antibodies (1 : 1000, cat. no. YZ-29450 N), monoclonal Cleaved caspase three antibodies (1 : 1000, cat. no. YZ-29450 N), and monoclonal Bcl-2 antibody (1 : 500, cat. no. YZ-12059U) purchased from Santa Cruz Biotechnology, Inc., Dallas, TX, USA, and monoclonal caspase-3 antibody (1 : 500, cat. no. ab19030Z) at four °C, and then incubated with HRP-conjugated secondary antibody (1 : 1000, Santa Cruz Inc., China, No. FLD4894-BK). Anti-GAPDH antibody (1 : 1000, Santa Cruz Inc., China, No. FLD4546-JM) was performed as an internal control. Prints were measured using the ECL substrate (Beyotime Inc., China).

### 2.9. Statistical Analysis

All experiments were performed independently, in triplicate. Quantitative variables were presented as the mean ± standard deviation (SD) from three independent experiments. As appropriate, the statistics were performed using the Student's *t*-test or one-way ANOVA in SPSS (version 17.0). A *P* value of <0.05 was considered statistically significant.

## 3. Results

### 3.1. Z-Ajoene Inhibits Cell Viability and Cell Proliferation in NSCLC Cells

We evaluated its cytotoxicity and how it affected behaviors of NSCLC A549 and H1299 cells after 72 h of Z-ajoene treatment using the CCK-8 assay. A significant dose-dependent (0–25 *μ*M) decrease in cell viability was observed in cells treated with Z-ajoene (Figure 1(b)), together with dose-dependent (0–10 *μ*M) inhibition of cell proliferation in colony formation and cell proliferation (Figures 1(c) and 1(d)).

### 3.2. Z-Ajoene Stimulates ERS in NSCLC Cells

**Figure 2:**
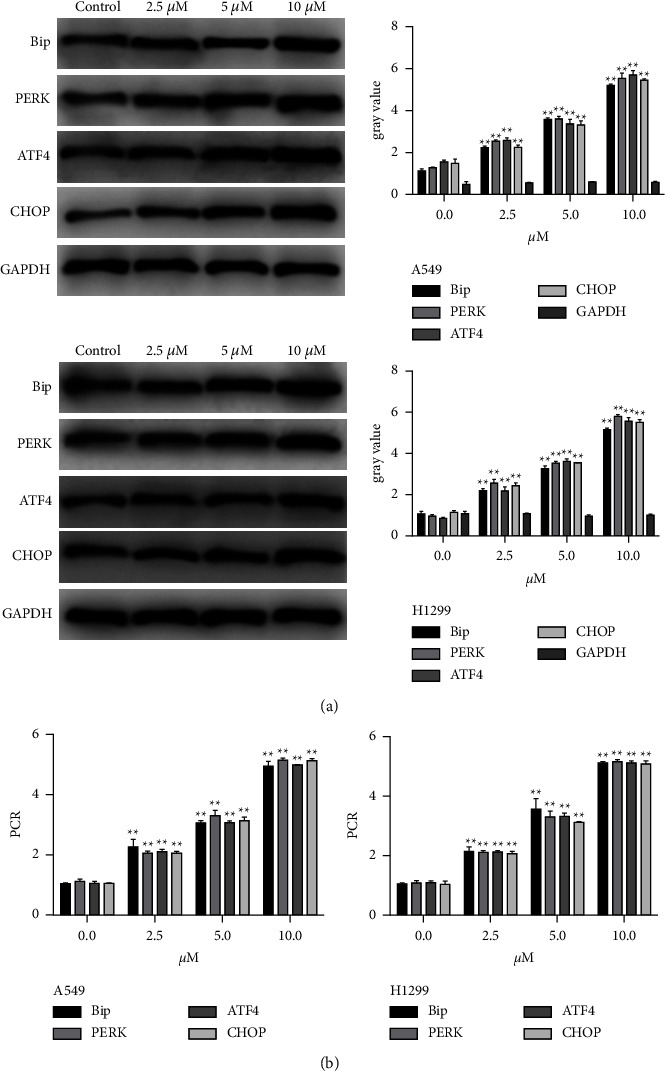
Z-Ajoene increases the expressions of ERR pathway components. (a) Significant increases in Bip, PERK, ATF4, and CHOP protein expressions were detectable in A549 or H1299 cells treated with Z-ajoene. (b) The same Bip, PERK, ATF4, and CHOP mRNA expression patterns were found in Z-ajoene treated cells.

Since Z-ajoene inhibited cell proliferation in NSCLC cells, we assessed whether Z-ajoene was involved in the ER stress pathway in NSCLC cells, and protein expressions of the pathway coSeminars in Interventional Radiologyponents were determined. The results showed pronouncedly increased expressions of binding protein (BiP) and protein kinase R-like endoplasmic reticulum kinase (PERK) after the Z-ajoene treatment (Figure 2(a)), as expected, which prompted us to detect the downstream UPR targets of PERK, activating transcription factor 4 (ATF4) and homologous protein (CHOP). ATF4 and CHOP proteins were significantly elevated with the Z-ajoene treatment compared to the control group. Z-ajoene was also very effective in inducing their expressions at the mRNA levels (Figure 2(b)).

### 3.3. Z-Ajoene Induces NSCLC Cell Apoptosis

**Figure 3:**
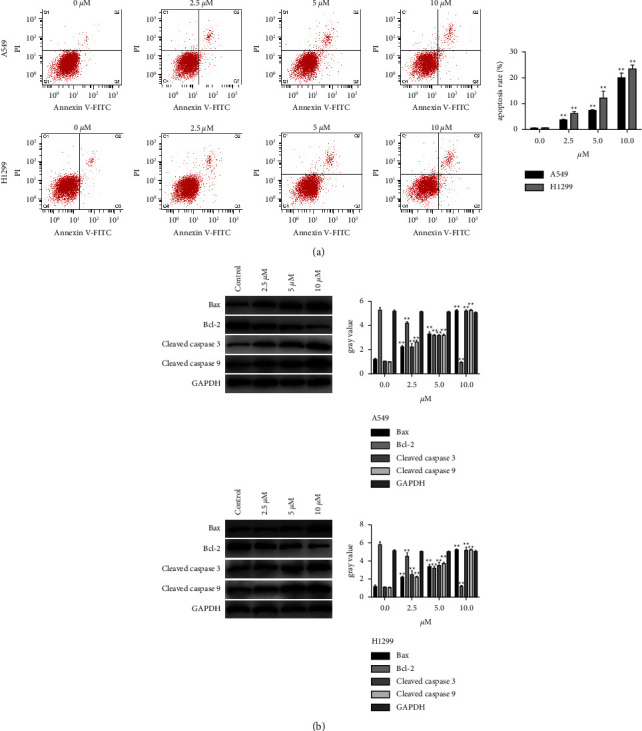
Z-Ajoene induces NSCLC cell apoptosis. (a) Enhanced cell apoptosis was detectable in Z-ajoene-treated cells, as indicated by flow cytometry. (b) Western blot showed increased expressions of pro-apoptotic Bax, cleaved caspase 3, and cleaved caspase 9 and a decreased expression of anti-apoptoticBcl-2 in Z-ajoene-treated cells.

Flow cytometry was performed to explore the effects of Z-ajoene on cell apoptosis in A549 and H1299 cells. Tumor cell apoptosis was markedly enhanced dose-dependently by the Z-ajoene treatment compared to that achieved in the control group (Figure 3(a)). The Western blot of several essential apoptosis-associated proteins showed that expressions of pro-apoptotic Bax, cleaved caspase 3, and cleaved caspase 9 expressions significantly increased in cells treated with Z-ajoene with a low expression of the anti-apoptosis protein Bcl-2dose-dependently (Figure 3(b)).

### 3.4. Z-Ajoene-Induced NSCLC Cell Apoptosis Is Associated with Enhanced ERS

**Figure 4:**
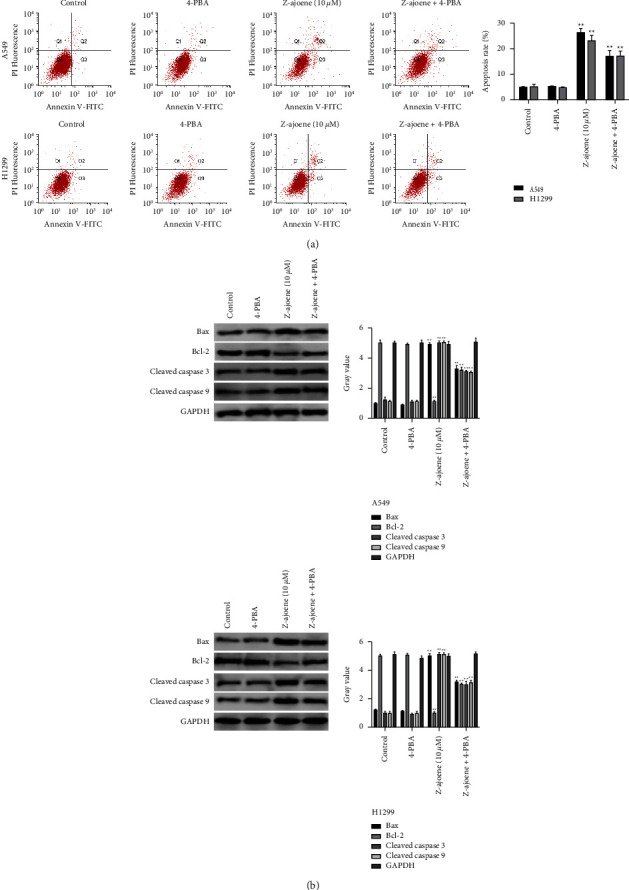
Z-Ajoene-induced NSCLC cell apoptosis is associated with enhanced ERS. (a) Flow cytometry revealed reduced apoptosis in cells co-treated with Z-ajoene and 4-PBA (an ERS inhibitor). (b) Western blot showed lower Bax, cleaved caspase 3, and cleaved caspase 9 expressions and higher Bcl-2 expression detected in cells cotreated with Z-ajoene and 4-PBA versus Z-ajoene-treated controls.

Z-Ajoene has stimulated apoptosis and ERS in NSCLC cells; however, whether there was a potential relationship between them is worthy of further exploration. We added 4-phenylbutyric acid (4-PBA), an ERS inhibitor, to detect whether apoptosis could be rescued. Compared to the Z-ajoene group, a decrease in cell apoptosis was observed in both A549 and H1299 cells cotreated with Z-ajoene and 4-PBA (Figure 4(a)). Similarly, pro-apoptotic Bax, Cleaved caspase 3, and Cleaved caspase nine expressions markedly decreased, and anti-apoptoticBcl-2 was elevated in the Z-ajoene + 4-PBA group versus Z-ajoene-treated controls (Figure 4(b)).

### 3.5. Z-Ajoene-Induced NSCLC Cell Apoptosis Is Activated by ERS Via DLG1/YAP Signaling Inhibition

**Figure 5:**
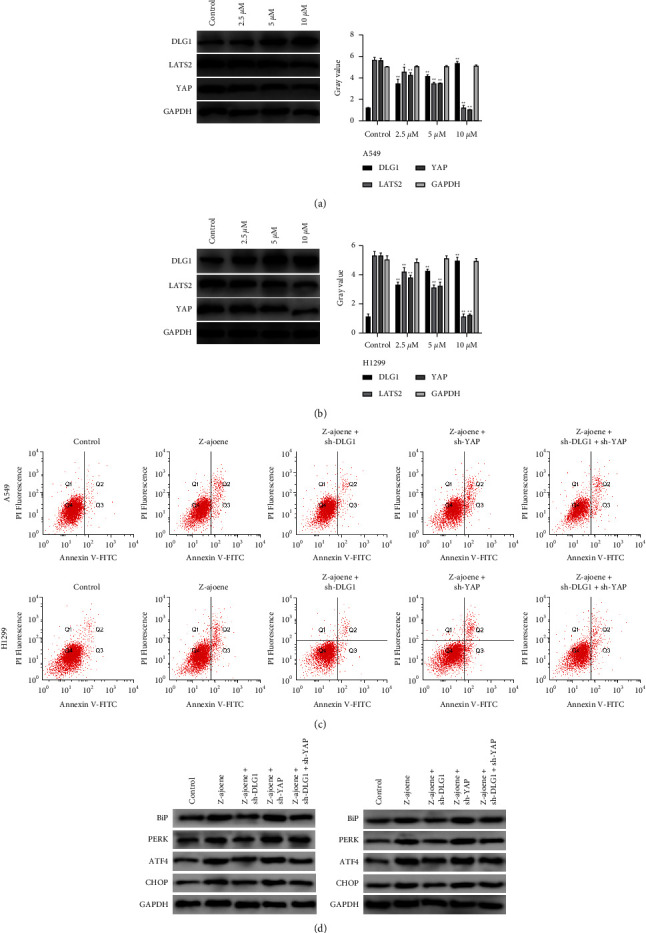
The pro-apoptotic effect of Z-Ajoene on NSCLC cell apoptosis is activated by ERS via DLG1/YAP signaling inhibition. (a) Western blot revealed DLG1, LATS2, and YAP protein expressions in A549 and H1299 cells treated with Z-ajoene. (b) The RT-qPCR assay revealed DLG1 and YAP mRNA expressions in A549 and H1299 cells. (c) The difference in cell apoptosis between groups was determined by flow cytometry. (d) Differences in Bip, PERK, ATF4, and CHOP protein expressions between groups were detected using the western blot analysis.

Furthermore, we assessed whether Z-ajoene exerted its pro-apoptotic effect on NSCLC cells via the DLG1/YAP pathway, an essential mediator of ERS. The Western blot assay showed that compared to the control group LATS2 and YAP protein expressions were significantly downregulated, and DLG1 levels upregulated dose-dependently in A549 and H1299 cells treated with Z-ajoene (Figure 5(a)).

A549 and H1299 cells were transfected with sh-DLG1 and/or sh-DLG1 with pcDNA YAP before the Z-ajoene treatment, and DLG1 mRNA expression was sharply downregulated in cells transfected with sh-DLG1, and YAP remarkably upregulated in pcDNA YAP-transfected cells (Figure 5(b)).  Figure 5(c) shows that the cells treated with Z-ajoene alone showed the strongest apoptosis. A reduced apoptosis rate was detectable in sh-DLG1-transfected cells, whereas the opposite finding was observed in sh-YAP-transfected cells. In cells co-transfected with sh-DLG1 and sh-YAP, the already inhibited apoptosis by sh-DLG1 was robustly reversed. We delineated that DLG1 knockdown remarkably suppressed Bip, PERK, ATF4, and CHOP expressions after the Z-ajoene treatment compared to negative controls, which were subsequently boosted after cotransfection with sh-YAP (Figure 5(d)).

## 4. Discussion

NSCLC is the most prevalent lung cancer and the primary cause of cancer-associated mortality worldwide [23, 24]. Some studies have proved the efficiency of existing treatments for nonsmall cell lung cancer, but the survival rate of advanced nonsmall cell lung cancer has not improved, which remains virtually unchanged. Therefore, the identification of novel and effective agents for the management of advanced NSCLC is the priority. In the present study, we found that Z-ajoenedose-dependently inhibited cell viability or cytotoxicity and proliferation in NSCLC cells, showing an antiproliferative effect against NSCLC.

The endoplasmic reticulum (ER) is an intricate organelle vital for cellular function and survival. When ER activity is hindered, the accumulation of unfolded proteins stimulates the transmembrane sensors to initiate the UPR, ultimately restoring ER homeostasis [25]. When the restoration of ER homeostasis or ERS attenuation fails, the UPR of ER activates signalings towards apoptosis. However, little research has been conducted on the specific mechanism of ER homeostasis recovery and focusing on ERS-mediated apoptosis may create new treatment options for cancer patients [26]. For the involvement of ERS in Z-ajoene-mediated apoptosis, our results revealed strong ERS responses to Z-ajoene treatment in A549 and H1299 cells, together with increased expressions of the ERS pathway components, BiP, PERK, ATF4, and CHOP. This finding demonstrates that the anticancer potential of Z-ajoene is triggered via ERS.

Apoptosis is strictly regulated by many proteins and pathways, of which the Bcl-2 family has a crucial role [27], including pro-apoptotic (Bax, Bim, Bcl-xs, Bak, Bid, Bad, and Bik) and anti-apoptotic (Bcl-2, Bcl-xl, Bcl-w, and A1) subfamilies [28]. Bax-type apoptotic proteins allow small molecules such as ions and cytochrome C to penetrate the mitochondrial membrane into the cytoplasm, leading to cell apoptosis. In our study, compared to untreated controls, Bax, caspase-3, and caspase-9 protein expressions were dose-dependently upregulated and anti-apoptoticBcl-2 downregulated with the Z-ajoene treatment, which supports an apoptotic effect of Z-ajoene against NSCLC A549 and H1299 cells.

DLG1 is a crucial component gene of the cell polarity module SCRIB-LGL-DLG. A negative correlation of DLG1 expression with YAP activity, or the inhibitory of DLG1 molecule on YAP activity, has been reported [29–31]. The Hippo/YAP signaling pathway plays an important role in cell growth regulation and organ size control during biological growth and development [32]. In the Hippo signaling, the upstream activator of the core effector YAP, LATS1/2, is stimulated by MST1/2-induced phosphorylation to boost YAP phosphorylation. Phosphorylated YAP is maintained in the cytoplasm, ubiquitinated, and degraded to make the downstream pathway of YAP in a static state, thus inhibiting YAP-mediated gene transcription. YAP degradation can be inhibited by oxidative stress, ischemia or hypoxia, physical factors, G protein-coupled receptors, or other signalings to increase YAP activity, allowing activated YAP to bind to TAZ and enter the nucleus to initiate relevant gene transcription [33]. Aberrant Hippo/Yap signaling may fuel cell proliferation and antagonize apoptosis for tumor occurrence and growth. In this study, Z-ajoene's regulation on YAP via DLG1 may be the mechanism responsible for its cytotoxic and apoptotic effects on NSCLC cells. Furthermore, our finding of LATS2 and YAP downregulation and DLG1 upregulation in a dose-dependent manner after the Z-ajoene treatment in A549 and H1299 cells is consistent with YAP regulation by DLG1, which was verified by YAP upregulation after DLG1 knockdown, together with a suppressed effect of Z-ajoene. Additional knockdown of the YAP gene reversed the effect of sh-DLG1 transfection into A549 and H1299 cells.

In summary, our study demonstrates that Z-ajoene induces ER stress through DLG1/YAP signal inhibition and promotes apoptosis in NSCLC cells. It is expected to offer more options for developing novel natural monotherapy or combination regimens for NSCLC.

## Data Availability

The data used to support the findings of this study are available from the corresponding author upon request.
